# Healthy Homes for Emigrants

**Published:** 1873-03

**Authors:** 


### HEALTHY HOMES FOR EMIGRANTS.

If a man wants to better his condition by removing to a new place, every care should be taken to secure two most indispensable points—

HEALTH AND A MARKET.

The land may be surprisingly productive, but if the cost of getting the produce to market is so great as to prevent its being sold at a profit, all the labor is lost; less productive lands near a market would yield more money. During the winter of 1872, corn was burned for fuel in some portions of the great Northwest, being cheaper than wood, for it was so far from market that it sold for ten cents a bushel. It is thus that thousands of farmers, owning rich lands, work hard for a lifetime, and find themselves at last

OLD AND POOR.

Others, again, open farms in fertile regions near railroads, or navigable streams, raise abundant crops, which being cheaply taken to market, yield great profits, and large wealth is accumulated in a few years; but in the meantime, the constitution has been broken down, the health hopelessly ruined from the effects of diseases peculiar to the locality.

In both these cases life was wasted, literally thrown away, spent in anxious, weary toil, under a multitude of privations and self-denials, from the one

FATAL MISTAKE

of not having located at healthy points, accessible to a market. With such sad facts in view, it is a great satisfaction to have found that a company of gentlemen have for some time been engaged in obtaining information and tabulating statistics in reference to the subject of procuring cheap lands, in healthy localities, near railroads and navigable waters, thus affording to individuals and families and colonies, the opportunity of obtaining homes, which, being healthy, productive, and profitable, should attract the attention of those who propose seeking their fortunes away from the home of their childhood.

